# Supplementary material for: Patterns of Intron Gain and Loss in Fungi
Source: PLoS Biol. 2004 Nov 30;2(12):e422. doi: 10.1371/journal.pbio.0020422 (PMC532390; doi:10.1371/journal.pbio.0020422)
Supplement: Table S1 — Also available at http://genes.mit.edu/NielsenEtAl/. (4.3 MB ZIP). [file pbio.0020422.st001.zip › NielsenEtAl/html/690.html]

AN0651.1.NCU06493.1.MG00365.1.FG05535.1


```
 CLUSTAL W (1.82) Multiple Sequence Alignments - Introns Inserted


Sequence 1: MG00365.1	353 aa
Sequence 2: FG05535.1	353 aa
Sequence 3: NCU06493.1	353 aa
Sequence 4: AN0651.1	353 aa
Alignment Length: 353 aa
Number Identitical Residues: 325 aa
Alignment Score (without introns) 12580


MG00365.1 	MGCGMSTEEKEGKARNEEIENQLKRDRLQQRNEIKMLLLG1AGESGKSTILKQMKLIHEG
NCU06493.1	MGCGMSTEEKEGKARNEEIENQLKRDRMQQRNEIKMLLLG1AGESGKSTILKQMKLIHEG
FG05535.1 	MGCGMSTEEKEGKARNEEIENQLKRDKMMQRNEIKMLLLG1AGESGKSTILKQMKLIHEG
AN0651.1  	MGCGMSTEDKEGKARNEEIENQLKRDKMMQRNEIKMLLLG1AGESGKSTILKQMKLIHEG
          	********:*****************:: *********** *******************

MG00365.1 	GYSRDERESFKEIIFSNTVQSMRVILEAMESLELPLEDQRMEYHVQTIFMQPAQIEGDVL
NCU06493.1	GYSRDERESFKEIIFSNTVQSMRVILEAMESLELPLADQRVEYHVQTIFMQPAQIEGDVL
FG05535.1 	GYSRDERESFKEIIFSNTVQSMRVILEAMESLELPLEDQRMEYHVQTIFMQPAQIEGDVL
AN0651.1  	GYSRDERESFKEIIYSNTVQSMRVILEAMESLELPLEDARNEYHVQTVFMQPAQIEGDSL
          	**************:********************* * * ******:********** *

MG00365.1 	PPEVGNAIEALWKDRGVQECFKRSREYQLNDSARY2YFDNIARIAAPDYMPNDQDVLRSR
NCU06493.1	PPEVGNAIEALWRDAGVQSCFKRSREYQLNDSARY2YFDNIARIAAPDYMPNDQDVLRSR
FG05535.1 	PPEVGSAIEALWKDRGVQECFKRSREYQLNDSARY2YFDNIARIAAPDYMPNDQDVLRSR
AN0651.1  	PSEVGNAIAALWQDAGVQECFKRSREYQLNDSAKY2YFDSIERIAQSDYLPTDQDVLRSR
          	*.***.** ***:* ***.**************:* ***.* *** .**:*.********

MG00365.1 	VKTTGITETTFIIGDLTYRMFDVGGQRSERKKWIHCFENVTTILFLVAISEYDQLLFEDE
NCU06493.1	VKTTGITETTFIIGDLTYRMFDVGGQRSERKKWIHCFENVTTILFLVAISEYDQLLFEDE
FG05535.1 	VKTTGITETTFIIGDLTYRMFDVGGQRSERKKWIHCFENVTTILFLVAISEYDQLLFEDE
AN0651.1  	VKTTGITETTFIIGDLTYRMFDVGGQRSERKKWIHCFENVTTILFLVAISEYDQLLFEDE
          	************************************************************

MG00365.1 	TVNRMQEALTLFDSICNSRWFIKTSIILFLNKIDRFKEKLPISPMKNYFPDYEGGDDYAA
NCU06493.1	TVNRMQEALTLFDSICNSRWFIKTSIILFLNKIDRFKEKLPVSPMKNYFPDYEGGDDYAA
FG05535.1 	TVNRMQEALTLFDSICNSRWFIKTSIILFLNKIDRFKEKLPVSPMKNYFPDYEGGDDYAA
AN0651.1  	TVNRMQEALTLFDSICNSRWFVKTSIILFLNKIDRFKEKLPVSPMKNYFPDYEGGADYAA
          	*********************:*******************:************* ****

MG00365.1 	ACDYILNRFVSLNQHETKQIYTHFTCATDTTQIRFVMAAVND1IIIQENLRLCGLI
NCU06493.1	ACDYILNRFVSLNQHETKQIYTHFTCATDTTQIRFVMAAVND1IIIQENLRLCGLI
FG05535.1 	ACDYILNRFVSLNQHETKQIYTHFTCATDTTQIRFVMAAVND1IIIQENLRLCGLI
AN0651.1  	ACDYILNRFVSLNQAEQKQIYTHFTCATDTTQIRFVMAAVND1IIIQENLRLCGLI
          	************** * ************************* *************
```
